# Stress in the workplace for healthcare professionals

**DOI:** 10.14814/phy2.14496

**Published:** 2020-07-06

**Authors:** Ian M. Williams, Wyn G. Lewis

**Affiliations:** ^1^ Department of Surgery University Hospital of Wales Cardiff UK

Workplace personal protective equipment (PPE) provision for healthcare professionals has recently received headline status, and rightly so, because thanks to the COVID‐19 pandemic lives are on the line. Such tools, by default, aim at providing physical protection against both visible and invisible threats to health, yet little work has been performed related to protection against arguably the most pervasive threat faced by healthcare professionals: stress.

Stress is a physical, mental, or emotional factor that causes bodily or mental tension. The stressors can be external (from the environment, psychological, or social situation) or internal (illness or, secondary to a medical procedure). Stress at work is common and not unique to one industry but a key unanswered question is how may it be measured and assimilated into reliable biometrics?

Biometrics at their best deliver data in three important domains. First, to help diagnose conditions (identifying early disease – diagnostic), second to forecast aggressive conditions (prognostic), and third to predict how well an individual will respond to treatment (predictive). The prime concern regarding stress is that if it goes unrecognized it may lead to burnout. Burnout is defined as a state of emotional, physical, and mental exhaustion caused by excessive and prolonged stress. The effects of burnout spill out not only into your professional job profiles but also into home and social life. Burnout is a gradual ongoing process taking months or years to manifest itself with many underlying lifestyles and personality traits, such as reluctance to delegate and tendency to perfectionism.

Initial methods of assessing stress included structured psychological surveys with the most commonly used assessment being the Maslach Burnout Inventory (MBI). The MBI consists of 22 questions, each scored from zero to six by the respondent, with zero indicating that the statement never occurs, and six indicating that it occurs daily. Scores are combined to provide overall values for each domain: Emotional Exhaustion (EE), Depersonalization (DP), and Personal Accomplishment (PA) (Masalach, Jackson, & Leiter, 2017). High levels of EE and DP but low levels of PA suggest burnout in each category. More recent interest has included assessment of physiological parameters including heart rate variability and measuring biological markers such as cortisol (both in blood and hair) (Arora et al., 2009).

The development of wearable biosensors permits continuous monitoring of heart rate variability as an indirect measurement of stress. The study by Hopkins et al. (2020) attempts to validate a consumer‐grade biosensor as a reliable and reproducible way of recording stress and placing a numerical value on it. Although only 12 participants were assessed there was a good assessment of energy expenditure and respiration in induced stressful environments. It certainly shows promise for future studies. These early devices may in future provide continuous monitoring of physiological parameters to provide a ratio whereby a stress reading might be calculated. This will invariably need to be evaluated in combination with a scoring system such as the MBI.

Stress in any workplace setting is dangerous because of its tendency to smoulder and become both harsh and chronic. Whilst even acute and short lived stress is clearly detrimental to the quality of life of the individual it may also lead to anxiety, depression, and even suicide if left untreated. For the healthcare professional and in particular surgeons, stress can affect both the technical and nontechnical performance, potentially leading to malpractice claims and may even lead to the surgeon changing practice, adopting an extreme risk averse approach to the detriment of patient care. Fellow colleagues frequently inflame the situation, alleging a loss of confidence or “loss of bottle” which may in turn lead to the public becoming less willing to seek an opinion or be treated by a doctor perceived to have a weakness particularly in insurance based practices.

How long does an individual need to be exposed to stress before he/she develops symptoms and signs referable to this? Principal contemporary hospital stressors seem to be the uncertainty regarding daily work patterns particularly those that are unpredictable, ambiguous or unfamiliar (Michie, 2002). With the modern organization of on call team little responsibility is delegated to post graduate year one doctors because during any given shift system as many as four more senior doctors are available for advice. However, during the normal working week outside of these perceived busy on‐calls they may only have direct contact and guidance at the beginning and end of the shift. This may leave them feeling less supported and having to take a greater degree of responsibility regarding clinical decisions. Hence, from the very first day of clinical work “burnout“ stimulus may begin. In contrast, Weenk et al. (2018), focused on the clinical tasks in a cohort of senior surgeons. For these individuals, as many would hypothesize there was more stress in the clinical operating theatre environment than in ward work or the outpatient setting.

The very nature of the job means an individual surgeon's actions have the potential to lead to serious harm or even death of a patient. Despite a robust consenting process and involvement of close family members in discussions prior to surgery, any complications post‐surgery can be a cause of significant stress (Shanafelt et al., 2010). Finding coping mechanisms to avoid these events is vital given that nowadays external reporting of short and long‐term outcomes following surgery is becoming an expected process easily obtained by the general public. Unfortunately, the difference between a procedural complication and a surgical error leading to patient harm is the difference between being liable to litigation or not (Turner et al., 2016). This correlation between stress and clinical error is not unique to surgeons. A 2018 study from the USA investigated this relationship and found that 10.5% of US physicians admitted to a medical error in the previous three months. Those physicians suffering symptoms akin to burnout, fatigue, or reporting suicidal ideation are significantly more likely to report such mistakes (Lebares et al., 2020).

The status of the UK as a first world nation comes at a price as the 24/7 pace of life comes with pressure to complete tasks to schedule and hit arbitrary targets in often very tight time limits. Surgery, as an occupation, is chaotic and indeterminate with many attributes unquantifiable such as communication, competence, and compassion. Often crude morbidity and mortality rates are the only measures to determine how well a doctor is performing at work. There is a delusion that all complexity at work can be rationalized and problems can be resolved by simple numerical analysis (O’Mahoney, 2017). However, work place issues are much more complicated and by adopting a gaming philosophy, a blame free environment is cultured and adversaries looked upon as worthy rivals for the benefit of all. New pathways to enable this would invariably reduce the stress and burnout rates from current levels.

Although advanced measures for recording stress are becoming more widely available in the form of consumer based products such as wearable technology, once identified there is limited evidence supporting effective interventions to combat it. Mindfulness training in the form of Mindfulness Based Stress Reduction (MBSR) shows promise and aims to increase an individual's resilience to stress. Lebares et al. (2018) at the University of California, San Francisco are currently performing a clinical trial examining the effect of MBSR in surgical interns. Doctors randomized to MBSR receive weekly 2‐hr classes and are suggested to perform 20 min of daily practice at home for a 8 weeks. These results could inform changes to postgraduate medical training and prove a necessary intervention for all medical professionals which will not only improve their well‐being but also the quality of care patients receive.

In conclusion, awareness of the underlying problem is critical and in the future it is likely to assume greater importance not least of which concerns the employer expectations in an environment of fewer hours and less employees. There are a number of methods for detecting stress, including survey methodology as well as invasive and physiological assessments. These must be incorporated and developed with the use of biosensors showing promise in this regard. There is still work to be done to find an effective intervention to prevent both burnout and improve the psychological well‐being of medical professionals. However, should the early promise demonstrated with the use of MBSR continue to produce positive outcomes then using the aforementioned techniques to detect people at risk of burn out in stressful environments would enable early intervention in order to stop and reverse worsening of a curable condition.
